# Independent Tailoring of Dose and Drug Release via a Modularized Product Design Concept for Mass Customization

**DOI:** 10.3390/pharmaceutics12080771

**Published:** 2020-08-14

**Authors:** Rydvikha Govender, Susanna Abrahmsén-Alami, Anette Larsson, Anders Borde, Alexander Liljeblad, Staffan Folestad

**Affiliations:** 1 Oral Product Development, Pharmaceutical Technology and Development, Operations, AstraZeneca, SE-43183 Gothenburg, Sweden; Susanna.Abrahmsen-Alami@astrazeneca.com (S.A.-A.); Anders.Borde@astrazeneca.com (A.B.); Alexander.Liljeblad@astrazeneca.com (A.L.); 2 Pharmaceutical Technology, Chemistry and Chemical Engineering, Chalmers University of Technology, SE-41296 Gothenburg, Sweden; anette.larsson@chalmers.se; 3 Innovation Strategies and External Liaison, Pharmaceutical Technology and Development, Operations, AstraZeneca, SE-43183 Gothenburg, Sweden; Staffan.Folestad@astrazeneca.com

**Keywords:** modular dosage form, reconfiguration, product variety, oral controlled release, melt-based processing, individualized therapy

## Abstract

Independent individualization of multiple product attributes, such as dose and drug release, is a crucial overarching requirement of pharmaceutical products for individualized therapy as is the unified integration of individualized product design with the processes and production that drive patient access to such therapy. Individualization intrinsically demands a marked increase in the number of product variants to suit smaller, more stratified patient populations. One established design strategy to provide enhanced product variety is product modularization. Despite existing customized and/or modular product design concepts, multifunctional individualization in an integrated manner is still strikingly absent in pharma. Consequently, this study aims to demonstrate multifunctional individualization through a modular product design capable of providing an increased variety of release profiles independent of dose and dosage form size. To further exhibit that increased product variety is attainable even with a low degree of product modularity, the modular design was based upon a fixed target dosage form size of approximately 200 mm^3^ comprising two modules, approximately 100 mm^3^ each. Each module contained a melt-extruded and molded formulation of 40% *w*/*w* metoprolol succinate in a PEG1500 and Kollidon^®^ VA64 erodible hydrophilic matrix surrounded by polylactic acid and/or polyvinyl acetate as additional release rate-controlling polymers. Drug release testing confirmed the generation of predictable, combined drug release kinetics for dosage forms, independent of dose, based on a product’s constituent modules and enhanced product variety through a minimum of six dosage form release profiles from only three module variants. Based on these initial results, the potential of the reconfigurable modular product design concept is discussed for unified integration into a pharmaceutical mass customization/mass personalization context.

## 1. Introduction

The provision of individualized pharmaceutical therapy, which ultimately aims to elicit optimal therapeutic outcomes a priori from all patients, requires the integration of key patient attributes into the design and manufacture of pharmaceutical products [1,2,3,4,5,6,7,8,9,10]. Recently, we presented a patient-centric framework of design requirements for individualized pharmaceutical products [3]. This was founded on the integration of key patient attributes and drivers for individualized therapy across three primary dimensions (biological, behavioral, and environmental) and a co-dimension (patient preference) into the design of pharmaceutical products, based upon a critical review of underpinning patient needs. Such integration reveals that simultaneous and independent individualization of multiple product features, such as the dose and drug release functionalities, in a controlled and predictable manner, is critical to tailoring products to fully meet the needs of all individuals [3].

As target patient segments become progressively smaller, with each segment having unique needs from the pharmaceutical product, enabling simultaneous and independent individualization of multiple product features, i.e., multifunctional individualization, will demand a marked increase in the number of product variants. This encompasses, for example, simultaneous provision of individualized dosing [3,11,12,13,14,15] and individualized drug release [3,16,17,18,19,20], where required [3]. Individualized dosing demands an increased number of dose strengths within a pre-established dose range. Similarly, individualized drug release demands an increased number of unique formulation-driven release profiles for a given active pharmaceutical ingredient (API) to enable robust individualized in vivo drug release and uptake and/or synchronized administration of multiple drugs [3]. However, under the current mass production paradigm, which is characterized by economies of scale, the provision of an increased number of product variants is constrained by limits in technical realizability and economic feasibility at low volumes [3,21,22,23]. Therefore, a shift towards alternative production approaches, such as mass customization/mass personalization [24,25,26,27,28], characterized by economies of scope, becomes critical to meet this product variety–volume complexity challenge and gain patient and societal benefits from individualized therapies [2,3]. In non-pharmaceutical branches of industry, established mass customization concepts like process flexibility, product modularity, and postponement (i.e., delayed point of product differentiation in the production chain) lend themselves to efficient management of product variety [3,29,30,31]. However, with the exception of process flexibility as a means to extend the manufacturing platform capability [32,33,34,35], such concepts remain inadequately addressed in a pharmaceutical context. For pharmaceutical products, providing the enhanced product variety necessary to fulfill the abovementioned individualized dose and individualized drug release requirements will rely on unified integration of these mass customization principles into both the design of the product and its manufacture. In this way, the required variety can be technically delivered alongside affordability and efficiency at smaller volumes. So far, although product design opportunities for individualization have been demonstrated, their holistic integration with a processing and production context for the provision of affordable individualized therapy is strikingly absent.

The aim of our study is to demonstrate multifunctional individualization by modular dosage forms through a product design concept capable of providing an increased variety of release profiles independent of the dose and size of the dosage form. In doing so, with a focus on the product platform and specifically on product modularity as an approach to provide affordable variety [31,36], this work explores and provides a basis for bridging the gap between established theoretical concepts of mass customization/mass personalization and eventual application in a pharmaceutical context.

### 1.1. Theoretical Considerations: Pharmaceutical Product Modularization

**Figure 1:**
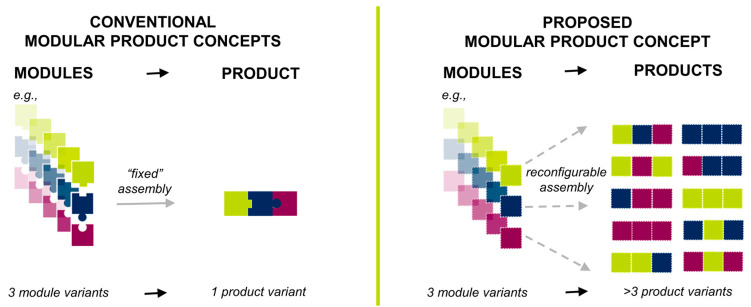
Difference between conventional modular pharmaceutical product concepts and a proposed modular pharmaceutical product concept highlighting the potential of the latter to promote affordable variety for the provision of individualized therapy through a unified product design-process-production approach to modularization.

Modular products have been defined previously as that which comprises building blocks or modules [37], whereby each module delivers a unique function required for the product to perform as intended [37,38]. These modules in turn consist of even smaller building blocks from which the module is constructed. For the purpose of this article, these distinctions are denoted, in order of decreasing size, as product (dosage form) > module > component. Modular pharmaceutical product archetypes already exist both on the market and in academic research [17,39,40,41,42,43,44,45,46,47,48,49,50,51,52,53,54,55,56,57], including, for example, granules, pellets, mini tablets, layered dosage forms, and compartmentalized structures, as well as the more recently demonstrated combined API and flexible dose product architectures [22,52,58]. However, these are currently designed to either promote process flexibility within the currently dominant mass production paradigm or potentially product variety within a full customization context. Figure 1 highlights the contrast between conventional modular product concepts and the proposed modular product concept.

Notably, Figure 1 exemplifies that an equivalent number of module variants in the conventional and proposed concept yields different degrees of product variety (one product variant vs. > three product variants in the example shown), with greater product variety accessible with the proposed modular product concept. This is due to the fact that conventional modular product archetypes are usually constructed by fixed assembly of modules into products [17,40,53,59,60,61]. Variety in the product offering is typically attainable through iterative modifications of a single initial product design until arriving at the variant of choice. Consequently, a greater number of module variants are required to generate a greater number of product variants. The drawback with this approach is that approaching lower volumes and higher variety of products is accompanied by a loss of economies of scale and an inevitable trade-off between affordability and variety [23]. This explains why most demonstrated oral dosage form modularization, even when some variety is present, still either exhibits interdependent dosage form size, dose, and drug release kinetics and/or a choice between fixed dose and fixed drug release kinetics at a given dosage form size [17,52,58,59,62,63,64,65,66,67]. From a product variety management perspective, conventional modular concepts are therefore limited in their ability to simultaneously deliver both the high variety and affordability necessary for individualization.

Recently, Siiskonen et al. have developed a customized product concept for modular pharmaceutical products to address the requirement for enhanced product variety in the provision of individualized therapy [68]. Building on the potential of this concept, which showed that 100 dose strengths were attainable from only five module variants, our study extends the exploration of modular product concepts towards multifunctional individualization, specifically for the independent provision of an individualized dose and individualized drug release. Through reconfigurable assembly (reconfiguration), our proposed concept explores the opportunity to realize a multitude of product variants whilst supporting affordability (Figure 1). Reconfigurable assembly of modules into products enables affordable variety by allowing a greater number of product variants to be generated from relatively fewer module variants [69]. Unlike conventional modular concepts, where reconfiguration is typically absent, product variety achieved through reconfiguration does not solely depend on module variety. The product variant of choice for an individual is instead selected from a set of multiple initial product designs. Provided that the concurrently developed product designs are based on an appropriate balance between standardization and differentiation in their features, both variety and affordability may be achieved [31,36], effectively achieving economies of scope almost on par with economies of scale.

### 1.2. Design of the Proposed Product Concept

**Figure 2:**
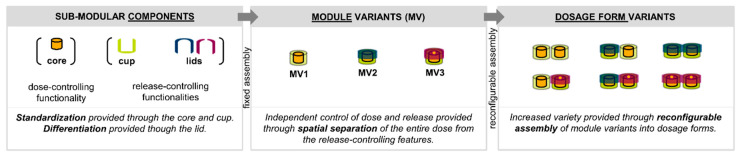
Proposed product concept for independent control of dose and drug release exemplifying the use of composite, reconfigurable drug release to increase release profile variety.

Figure 2 illustrates key design features of the proposed modular product concept to provide independent control of dose and drug release demonstrated through an increased variety of drug release profiles in the product offering.

The product was designed as a solid oral dosage form with a modular architecture. For the purpose of this study, a dosage form was assumed to comprise two out of three possible module variants, i.e., a low degree of product modularity. These three module variants were designed to provide unique drug release profiles but contain the same dose. A key feature for simultaneous and independent tailoring of the dose and drug release to an individual is the spatial separation of the dose- and release-controlling functionalities in the module variants. Reconfigurable assembly of these module variants into dosage forms, combining either two identical or two dissimilar module variants, was designed to increase variety in resulting in vitro drug release profiles on the dosage form level for a single API-containing drug product, without needing to increase the number of module variants. Importantly, it was only through one component (i.e., the lid) that variety in the release kinetics of the module variants was introduced whilst other components (i.e., the core and cup) remained fixed. Altogether, spatial separation of features of interest supports multifunctionality, whereas reconfigurable assembly of module variants into dosage forms and a balance between standardization and differentiation of submodular components promote affordable variety. These characteristics therefore underpin the suitability of this concept for tackling the product variety–volume complexity challenge.

Our work proposes and constitutes, to the best of the authors’ knowledge, a first demonstration of a product concept that supports multifunctional individualization whilst also meeting the challenge from enhanced product variety management. Except for an early recommendation in our previous work [3], for the purpose of affordable variety for individualized therapy, neither multifunctional individualization nor the provision of an increased variety of unique release profiles in a controlled and predictable manner through reconfiguration have been suggested or demonstrated yet.

The realization of the proposed concept is based on the hypothesis that, in the absence of interactions between module variants, module variants with independently controlled dose- and drug release-determining functionalities can contribute predictably and independently to the net dose and drug release functionalities of the dosage form they construct.

Although co-development of product and manufacturing platforms in a mass customization context is crucial to support enhanced pharmaceutical product variety, especially at lower volumes, for the purpose of demonstrating our proposed concept, the product platform (with specific focus on solid oral dosage forms) is considered within the scope whereas manufacturing platforms, beyond their technical ability to prototype with the intended product performance, are considered out of scope. Since manufacturing networks are out of scope, scale-up and dedicated stability testing under varying conditions relevant to storage and transport are also beyond the scope of this study.

## 2. Materials and Methods

### 2.1. Materials

Metoprolol succinate (MS; MW 652.8 g/mol) was obtained from AstraZeneca (Gothenburg, Sweden). Vinylpyrrolidone-vinyl acetate copolymer (VA64; Kollidon^®^ VA64) was supplied by BASF (Ludwigshafen, Germany). Polyethylene glycol 1500 (PEG1500; MW 1500 g/mol), sodium phosphate dibasic dihydrate (MW 177.99 g/mol, assay ≥ 98.0%) and sodium phosphate monobasic monohydrate (137.99 g/mol, assay ≥ 99.0%) were purchased from Sigma-Aldrich Chemie GmbH (Steinheim, Germany). ZMorph polylactic acid (PLA) filament (white, 1.75 mm diameter) was obtained from ZMorph S.A. (Wroclaw, Poland) and Aquasolve™ polyvinyl acetate (PVA) filament (natural, 1.75 mm diameter) was purchased from Formfutura BV (Nijmegen, Netherlands).

### 2.2. Methods

#### 2.2.1. Technical Realization of the Product Concept

The module variants were constructed from submodular components whose structural design and material selection provided each module variant with its respective dose and drug release kinetics. The dose-controlling functionality of each module variant was derived from a standardized drug-containing rapidly erodible polymeric core, intended to convey an identical dose to each module. Two release-controlling submodular components were designed, one was standardized between module variants (cup) and the other was customized for each module variant (lid). The standardized water-insoluble PLA cup enclosed the bottom and sides of the core, allowing the surface area available for drug release to remain constant during dissolution. The presence and/or type of lid provided the module variants with their distinct drug release kinetics. Module variant 1 (MV1) with no lid was designed to provide rapid drug release, module variant 2 (MV2) with a water-soluble lid of predefined thickness was designed to generate a lag phase followed by rapid drug release from the core, and module variant 3 (MV3) with a water-insoluble lid with a central orifice was designed to provide slower initial drug release kinetics through the reduced area available for initial hydration of the core.

#### 2.2.2. Fused Deposition Modeling (FDM)

**Figure 3:**
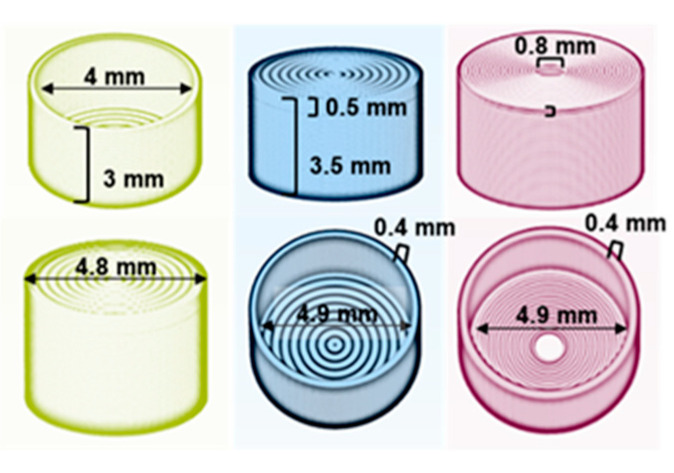
Key feature dimensions for fused deposition modeling (FDM)-printed submodular components, i.e., cup (left), soluble lid (middle), and insoluble lid (right), viewed upright (top row) and inverted (bottom row).

Fused deposition modeling (FDM) was used to fabricate the cup and lid submodular components. Digital models of the cup and lid were created as ‘.stl’ files using Autodesk^®^ TinkerCAD™ (Autodesk, Inc., San Rafael, CA, USA). The generated ‘.stl’ file was subsequently imported into Simplify3D^®^ (version 4.1.1., Simplify3D LLC, Cincinnati, OH, USA) for control of printing parameters and subsequent printing. Critical cup and lid model dimensions are described in Figure 3.

A ZMorph VX multitool 3D printer (ZMorph S.A., Wroclaw, Poland), equipped with a single 0.3 mm diameter extrusion nozzle, was used to independently print the cup and lid submodular components from PLA and PVA filaments, respectively, for downstream assembly. The lids were designed with overhanging side walls and the cups and lids were all printed with matching 0.1 mm layer heights to facilitate interlocking and adhesion and prevent dislodging of the lids once the modules were assembled. Prior to the printing of each component, the build platform was leveled and heated to 60 °C to facilitate adhesion of the structures onto the platform. The set temperature for the nozzle was 200 °C, for both PLA cups and PVA lids.

#### 2.2.3. Melt Extrusion of the Drug-Containing Filament

Hot melt extrusion (HME) was the first step in core fabrication and was selected in order to obtain a homogeneous distribution of the drug in a carrier via HME’s dispersive and distributive mixing. A formulation containing 40:20:40% *w*/*w* MS:PEG1500:VA64 was prepared by HME. This drug concentration allowed a dose of approximately 25 mg MS (lower limit of marketed dose) to be incorporated into each dosage form. The incorporation of both low molecular weight PEG and VA64 as carriers facilitated the melt molding and melt extrusion processes, respectively, whilst promoting rapid drug release. MS powder, PEG1500, and VA64 powder were weighed in a weigh boat in a 2.5 g batch size and mixed with a spatula until homogeneous upon visual inspection. HME was performed using a 5 mL capacity Xplore micro compounder (Xplore Instruments BV, Sittard, The Netherlands), affixed with conical mixing screws and a circular die, 1.5 mm in diameter. The physical mixtures were fed via a hopper into the barrel maintained at a constant temperature profile of 80 °C and a screw speed of 50 rpm during feeding and recirculation. This processing temperature was sufficiently low to allow consistent feeding into the barrel without particle bridging in the hopper and an appropriate material viscosity for consistent extrusion through the die and sufficiently high to operate within the motor load (torque) limit of the extruder at the chosen batch size. After complete feeding (˂ 1 min), mixtures were recirculated for 10 min to aid homogenization prior to extrusion through the die to obtain a cylindrical filament, which was allowed to cool at room temperature. Ejection was force-controlled at 100 N until a maximum screw speed of 400 rpm was reached. Filaments were stored in sealed plastic bags at room temperature prior to further processing.

#### 2.2.4. Melt Molding of Drug-Containing Cores

Melt molding was the second step in core fabrication. Empty PLA cups fabricated by FDM were weighed in a Mettler MT5 analytical balance (Mettler Toledo, Greifensee, Zurich, Switzerland). Melt extruded filaments were cut with a blade and manually filled into each PLA cup. The cups with unmelted filaments were placed in an oven (Memmert GmbH + Co.KG, Schwabach, Germany) at 100 °C for 15 min, the shortest duration required to achieve molding at this temperature. A higher temperature was required during melt molding than melt extrusion due to the absence of shear stress or applied force from rotating screws in the former process. This melt molding temperature allowed the molten formulation to fill the cup whilst the surrounding PLA cup maintained its structural integrity. The selection of a melt molding process for core fabrication allowed the core formulation to form a good seal with the inner bottom and sides of the surrounding PLA cup. In the absence of a lid, the exposed surface area for subsequent hydration and drug release would be identical in all module variants. The cups for MV1 and MV2 were filled completely in 2 sequential additions of 15 min each whereas the cups for MV3 were overfilled in 3 sequential additions of 15 min each to prevent formation of an air pocket beneath the MV3 lid orifice. The core-containing cups were weighed, and the mass of the cores calculated. Filled cups were stored in closed plastic well plates at room temperature prior to analysis.

#### 2.2.5. Thermal Characterization

Thermogravimetric analysis (TGA) using a TGA/DSC 3+ STARe system instrument (Mettler Toledo, Greifensee, Zurich, Switzerland) was performed on raw materials to determine the onset of thermal degradation (T_deg_). Materials were weighed in open 100 µL aluminium crucibles and heated from 30 to 500 °C at 10 K/min under a nitrogen atmosphere set at a flow rate of 50 mL/min. T_deg_ was reported as the first observed deflection (mass loss) from the initial baseline in the weight vs. temperature curve in the absence of water loss. Glass transition temperatures (T_g_) and/or melting points (T_m_) of the raw materials and melt extruded filaments were probed between room temperature and below T_deg_ of the drug, which represents the range of storage and processing conditions for the materials. T_g_ of raw materials below room temperature (MS and PEG1500) were obtained from existing literature. These thermal events were measured by differential scanning calorimetry (DSC) in a DSC 2 STARe system instrument (Mettler Toledo, Greifensee, Zurich, Switzerland). The samples were weighed in 40 µL aluminium crucibles, which were sealed by lids with a pinhole for subsequent analysis. The instrument was run in a heat-cool-heat cycle at 10 K/min under a 50 mL/min nitrogen atmosphere from 25 to 170 °C. STARe software (version 16.00b, Mettler Toledo, Greifensee, Zurich, Switzerland) was used for instrument control and subsequent analysis of thermograms. T_g_ values were determined at the midpoint of the T_g_ range and T_m_ at the peak of the melting endotherm. Furthermore, the percentage crystallinity of MS in the melt extruded filament was calculated by normalizing the melting enthalpy of MS in the melt extruded filament to the melting enthalpy of the pure MS powder (100% crystallinity) and to a nominal MS content of 40% w/w in the filament. All samples were prepared in triplicate for analysis.

#### 2.2.6. X-Ray Computed Microtomography (X-Ray µCT)

Non-destructive visualization of the module variants was performed using X-ray computed microtomography (X-ray µCT). The assembled module variants comprising the cup, core, and affixed lid (for MV2 and MV3) were analyzed intact using a high-resolution SkyScan 1272 instrument (Bruker, Antwerp, Belgium), with a 50 kV, 181 µA X-ray source and a charge-coupled device (CCD) detector fiber-optically coupled to a scintillator. SkyScan 1272 control software (v1.1.17, Bruker microCT, Antwerp, Belgium) was used for instrument operation. Samples were imaged in batches of 4 at a pixel size of 19.8 µm or smaller. Three-dimensional imaging was achieved by rotation through 180° with steps of 0.5° averaging 2 frames per position, with 3 × 3 binning, resulting in a resolution of 1344 pixels × 896 pixels and a total acquisition time of 23 min per batch. Image reconstruction was performed from a batch of 4 objects using NRecon (v1.6.10.2, Bruker, Antwerp, Belgium) at a resolution of 19.8 µm, adjusting misalignment, reducing ring artifacts, and applying beam hardening correction (10%). During reconstruction, the subscans were fused in the z-direction and both x/y shift correction and rotation correction were applied. DataViewer (v1.5.6.2, Bruker microCT, Antwerp, Belgium) was used for data analysis. Core diameters for all module variants, lid thicknesses for MV2, and orifice sizes for MV3 were obtained from DataViewer. The orifice diameter was measured for a 0.4 mm orifice size although final drug release tests were conducted on MV3 with a 0.8 mm orifice size. A digital caliper set at 0.8 mm was used to verify that 0.8 mm orifices were consistently printed.

#### 2.2.7. In Vitro Drug Release and Drug Content Homogeneity

In vitro drug release testing of MS from the module variants, both alone and in combinations of two module variants (to represent the dosage form variants), was carried out in duplicate using a USP 2 dissolution apparatus (Varian 705-DS, Agilent Technologies, Santa Clara, CA, USA) with a suspended stainless-steel stationary basket (mesh size 8), which housed the sample. To prevent floating during dissolution, the module variants were glued to the metal flap supplied with the stationary basket using ethyl cyanoacrylate adhesive. The USP 2 apparatus was operated at 37 ± 1 °C, 50 rpm with 900 mL pH 6.8 phosphate buffer (I = 0.1 M) and was equipped with an autosampler (Agilent 8000 Dissolution Sampling Station, Agilent Technologies, Santa Clara, CA, USA) programmed to withdraw 1.5 mL sample aliquots at predefined time intervals without media replacement. Withdrawn aliquots were analyzed by ultra-performance liquid chromatography coupled with ultraviolet spectroscopy (UPLC-UV) to quantify MS concentration. For all drug release tests involving MV3, to eliminate the accumulation of air from the dissolution medium at the orifice of MV3, the dissolution medium was stirred for 2 h prior to commencement of the drug release test and the lid of the dissolution vessel was tapped gently during dissolution at 0 min and 15 min, respectively.

Prior to USP 2 dissolution of individual module variants and dosage form variants, preliminary investigations of drug release kinetics from cores only, MV1, and MV3, were performed in duplicate in 250 mL glass beakers containing 100 mL pH 6.8 phosphate buffer (I = 0.1 M), which maintained sink conditions (relative to crystalline MS solubility) for MS dissolution. The purpose of this preliminary release test was to guide selection of MV3 orifice size based on the orifice size that would provide distinct drug release kinetics compared to MV1 and which could therefore be used to demonstrate reconfigurability. Samples were suspended in the medium using a stainless steel stationary basket analogous to that described in the USP 2 dissolution setup. This smaller scale dissolution test was operated at room temperature with 500 rpm stirring with an Arex magnetic stirrer (VELP^®^ Scientifica, Usmate, Italy). At predefined time intervals, 1 mL aliquots were withdrawn. UV absorbance spectrophotometry was used to quantify MS absorbance at 275 nm and MS concentration was calculated from a calibration curve (concentration range 2–100 µg/mL). As an indication of homogeneity, MS content was also determined along the length of the melt extruded filament by dissolving 5 mm long filament sections (*n* = 10) weighing approximately 5 mg in 10 mL phosphate buffer and quantifying MS via UV absorbance as described above.

MS concentrations in the aliquots withdrawn from the USP 2 dissolution apparatus were quantified using a Waters Acquity UPLC system (Waters, Milford, MA, USA) featuring a binary solvent manager, column manager, and sample manager. The UPLC system was equipped with a reversed phase Acquity UPLC BEH C18 column (1.7 µm particle size, 2.1 mm inner diameter × 50 mm length) and an Acquity UPLC PDA detector operated at 275 nm (Waters, MA, USA). MS calibration standards were prepared ranging in concentration from 0.25 to 20 µg/mL for construction of a calibration curve and subsequent quantification of MS concentrations in samples. The % relative standard deviation (RSD) for all standard curves were below 5%. Of the standard or sample 10 uL was injected into a mobile phase pumped at 1 mL/min through the column, maintained at 40 °C, in an isocratic gradient of 75% water containing 0.03% trifluoroacetic acid and 25% acetonitrile containing 0.03% trifluoroacetic acid. Empower 3 Chromatography Data Software (Waters, Milford, MA, USA) was used for both instrument control and data analysis.

## 3. Results

### 3.1. Realization of the Product Concept

Functional realization of the product concept requires accurate and precise translation of the dose- and drug release-determining design features into the physical construct. Table 1 shows the measured mass and dimensions of the submodular components and/or specific features of interest for dose and drug release in MV1 (no lid), MV2 (water soluble lid), and MV3 (water insoluble lid with orifice).

MS content measured along the length of the melt extruded filament was 39.4% ± 1.3% *w*/*w*. With a % RSD of 3.2% in filament sections weighing approximately 5 mg, the filament was deemed homogeneous on an approximately 6-fold smaller length scale than that required for core fabrication. With this homogeneous distribution of MS in melt extruded filaments, the dose for MV1, MV2, and MV3 depended on reproducibility of both FDM-printed PLA cups and melt molded cores. FDM dispensing precision of the PLA for the cups was high with a 1.1% RSD in mass. In contrast, the somewhat lower precision in melt molded core mass of 3.6% and 4.2% RSD for normal filled cores (MV1 and MV2) and overfilled cores (MV3), respectively, reflects the reliance on manual sectioning and filling of extruded filaments into the PLA cup prior to melt molding, compared to the automated dispensing during FDM to generate the PLA cups. Regardless, core masses were within an acceptable 5% RSD in all cases, which, together with the filament drug content homogeneity, was expected to translate to acceptable precision in dose.

The drug release rate-determining functionality was designed as the presence and/or type of lid in each module variant given that the fastest drug release was provided by the core. This translated to core diameter precision (2.8% RSD), lid thickness precision (6.4% RSD), and orifice diameter precision (2.2% RSD) for MV1, MV2, and MV3. The dimensional data in Table 1 was obtained from X-ray µCT images. The pixel size was adequate for the individual feature measurements. Contributions to lid thickness variability, above the acceptable 5% RSD, is discussed in the X-ray µCT section below (Section 3.3). Additionally, core diameter precision was also critical for MV2 since the drug release rate after the water-soluble lid dissolves is dependent on the core diameter, analogous to MV1. For MV3, the orifice diameter precision was measured for a target orifice diameter of 0.4 mm, which had an acceptable 2.2% RSD in diameter. However, the final target orifice diameter for the MV3 constructs used in subsequent drug release testing was 0.8 mm. Previous studies have shown that FDM dispensing precision decreases with dispensing volume [70]. It can therefore be concluded that larger feature sizes are expected to be generated with higher precision during FDM. Therefore, the final 0.8 mm orifice precision is taken to be at least equivalent but expectedly higher than that of the 0.4 mm orifice.

### 3.2. Thermal Characterization

**Figure 4:**
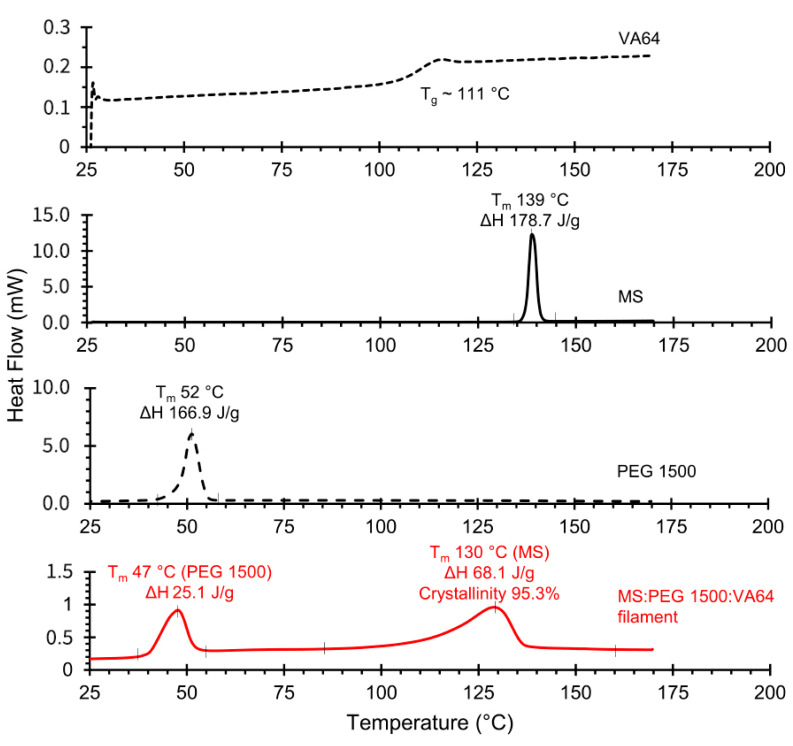
Differential scanning calorimetry (DSC) traces of melt-extruded drug-containing filament (red line) relative to raw materials included in the filament composition (black lines) whilst heating from 25 to 170 °C.

The cores are the dose-controlling features of all module variants and were melt molded from melt extruded drug-containing filaments. Figure 4 shows the thermal transitions, which occurred between 25 °C and 170 °C, of the raw materials for melt extrusion and the melt extruded filament (40% *w*/*w* MS: 20% *w*/*w* PEG1500: 40% *w*/*w* VA64).

Pure crystalline MS displayed a melting endotherm at 139 °C (T_m_, midpoint). In the melt extruded filament comprising 40% *w*/*w* MS: 20% *w*/*w* PEG1500, and 40% *w*/*w* VA64, melting endotherms were visible in the region of PEG1500 and MS. The melting endotherm corresponding to MS was observed in the melt extruded filament at a T_m_ of 130 °C. Comparing the melting enthalpies of pure crystalline MS with that of MS in the filament revealed a reduction in the melting enthalpy from 178.7 to 68.1 J/g (total weight). The melting enthalpy of pure crystalline MS is in agreement with that reported in the literature [71]. Reduced melting enthalpies in the filament are in part attributable to concentration reduction from 100% in the raw materials to 40% MS and 20% PEG1500 in the filament. However, this melting point depression was accompanied by a slight reduction in the degree of crystallinity from 100% to 95.3% in the filament, indicating conversion of some MS to the amorphous form and/or solubilization of MS in the carrier/s [72]. A similar phenomenon was observed in the region of PEG1500 in the filament resulting in a reduction in the degree of crystallinity of PEG1500. Indeed, reported Hansen solubility parameters of MS, PEG1500, and VA64 are similar at 21.7 MPa^1/2^ [73], 21.4 MPa^1/2^ [74], and 21.5 MPa^1/2^ [75], respectively, supporting solubilization. The T_g_ of MS and PEG1500 below the temperature range of interest in this study was reported as 2.2 °C [73] for MS and −42 °C [76] for PEG1500 in the existing literature. The T_g_ of VA64 was determined as approximately 111 °C determined from heat cycle 2. Previous literature corroborates the presence of a characteristic broad endothermic peak between 25 and 100 °C due to water loss in heat cycle 1 and therefore T_g_ determination of VA64 from heat cycle 2 [77]. All other DSC traces represent heat cycle 1. Despite the presence of crystalline MS in the melt extruded filament, drug content was found to be homogeneous along the length of the filament on a smaller length scale (approximately 5 mg filament) than that required for core construction (approximately 29 mg filament). Since MS has a high aqueous solubility and the module core was formulated as a rapidly eroding matrix, the relative contributions of amorphous and crystalline MS was not expected to significantly alter the drug release performance of the module variants. All processing, by FDM, HME, and melt molding, was performed below the degradation temperatures of the raw materials, which were measured as 176 °C (MS), 311 °C (PEG1500), 276 °C (VA64), 308 °C (PLA), and 270 °C (PVA).

### 3.3. X-Ray Computed Microtomography

**Figure 5:**
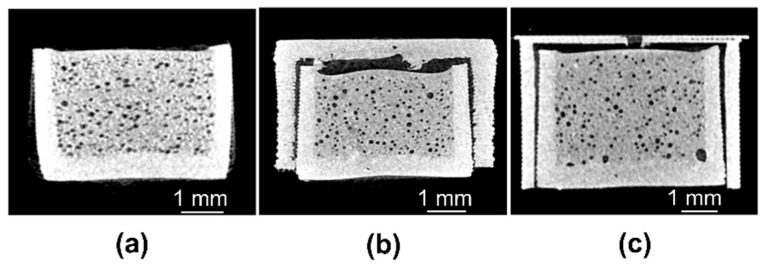
Computed microtomographs of longitudinal sections through the midline of: (**a**) MV1; (**b**) MV2; (**c**) MV3 with 0.4 mm orifice.

Figure 5 shows longitudinal sections through the center of MV1, MV2, and MV3 (with 0.4 mm orifice) and a cross section through MV2, revealing additional potential influences on drug release rates.

Figure 5 confirms that the cores were molded in direct contact with the inner walls and floor of the PLA cups. PLA cup integrity was also verified through the absence of any defects traversing the wall or floor of the PLA cup that could allow entry of dissolution media. Together, these results confirmed that, as designed, the core diameter is expected to be release rate determining for MV1 and for MV2 after lid dissolution. Figure 5 also shows that porosity was present in all cores after melt molding. Porosity has been observed previously in melt-extruded VA64 filaments [78]. However, unlike the applied force exerted by the screws during mixing in melt extrusion, we postulate that the melt molding process, which involved loose stacking of filament sections in the cup followed by melting without any applied force, was likely to contribute more to eventual core porosity. Three-dimensional image reconstruction revealed that these pores were not interconnected throughout the matrix. Figure 5b, which represents MV2, shows some sporadic over-deposition of PVA from the FDM nozzle in fabricating the lid, which could influence lag times once polymer swelling and dissolution proceeds. Figure 5b,c reveal that, in module variants with lids, cores with a normal fill volume created a headspace between the core and the lid. This headspace and core porosity are both sources of air within MV3, which could contribute to orifice obstruction or variable release kinetics [79]. Note that in final drug release tests involving MV3, this headspace was eliminated by overfilling of the PLA cup with the core. The gaps visible between the PLA cup walls and the lids were on the order of 200 µm or less at a fixed point, which did not translate to looseness of fit and allowed the lids to remain attached to the cup either until lid dissolution was complete (MV2) or throughout the drug release test (MV3).

### 3.4. In Vitro Drug Release

**Figure 6:**
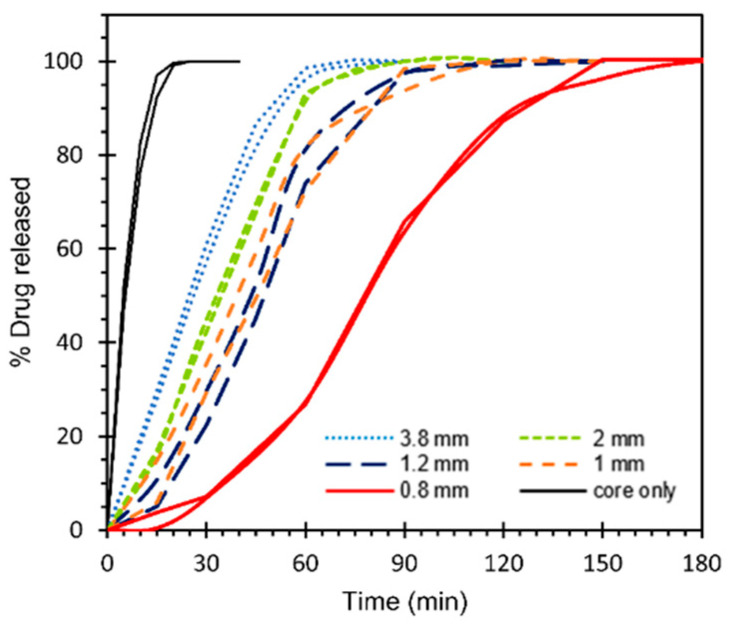
% Drug released vs. time for module variants with varying orifice size to determine the final orifice size for selection of MV3. The core only and MV1 with a 3.8 mm diameter exposed core face are presented as references. Data is presented as the minimum and maximum of *n* = 2 samples.

Drug release kinetics for modules with varying orifice size are shown in Figure 6 in order to aid final selection of the appropriate orifice size for MV3, which should display distinct drug release kinetics to MV1 and allow reconfigurability to be demonstrated in final drug release tests. Preliminary drug release testing in 250 mL media confirmed rapid and complete drug release from the free core with a T80 of 10 min (Figure 6).

A reduction in the drug-containing core dimensions with respect to time was observed with complete drug release evident once the entire core had dissolved. This was expected from a core matrix comprising a water-soluble drug in a water-soluble PEG1500 and VA64 matrix. According to the product design, rapid release from the core was intended to allow the release rate-limiting features to be surface area of the exposed core face, lid thickness, and orifice size for MV1, MV2, and MV3, respectively. As expected, Figure 6 shows that drug release from MV1 (3.8 mm diameter in Figure 6), with a T80 of 43 min, was indeed slower than that of the free core due to the reduced surface area available for hydration and subsequent drug dissolution. Figure 6 also illustrates drug release kinetics as a function of lid orifice size, which was used to govern MV3 selection. Drug release kinetics with progressively smaller orifice size revealed slower (T80 of 110 min) and mechanistically distinct release kinetics at an orifice size of 0.8 mm, whereby initial drug release increased with time, in contrast to the initial zero-order release exhibited by MV1. Compared to the 0.8 mm orifice size, orifice sizes ranging from 1–2 mm (T80 of 52-65 min) all had similar release kinetics to MV1 indicating that the contribution of initial hydration differences to the full release mechanism was not significant at this range of orifice sizes. Consequently, an orifice size of 0.8 mm was selected for MV3. MV2 was designed to be identical in release rate to MV1 after an initial lag period provided by the dissolution time of the water-soluble PVA lid. Although the thickness of the lid would influence the duration of the lag phase, a lag phase of any duration would differentiate MV2 release kinetics from that of MV1 and MV3. Consequently, no preliminary drug release tests were required for the selection of MV2.

**Figure 7:**
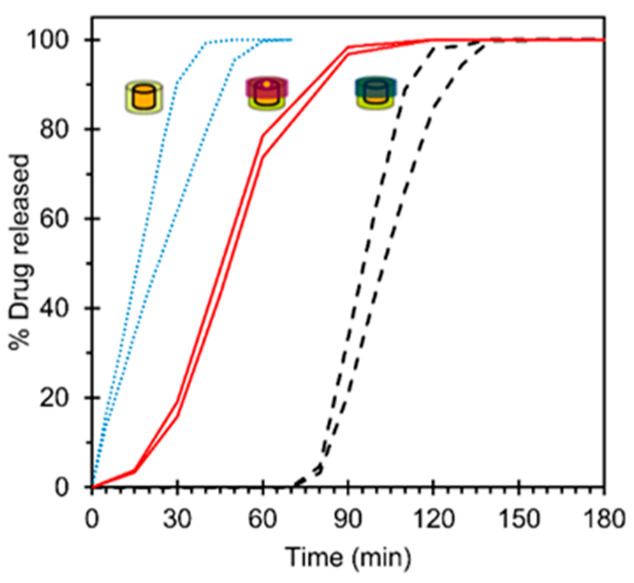
% Drug released vs. time for MV1 (blue), MV2 (black), and MV3 (red). Data is presented as the minimum and maximum of *n* = 2 samples.

Figure 7 shows drug release kinetics for the final selected individual module variants MV1, MV2, and MV3 under the USP 2 dissolution test conditions described in the methods section.

Three unique release profiles were obtained for the three module variants, with sufficient reproducibility such that they could be used as the basis for reconfiguration into the final dosage form variants. MV1 and MV2 exhibited mean T80 of 33 min and 110 min, respectively. Considering the 70 min lag phase during which the water-soluble PVA lid eroded, MV2 release kinetics after the lag phase were comparable to MV1, as designed, with mean T80 for MV2 achieved approximately 40 min after commencement of drug release. The lag phase for MV2 was provided by the lid swelling and dissolution prior to drug release. The initially slower drug release rate between 70 and 80 min, before the release rate reaches that of MV1, could be attributed to incomplete dissolution of the PVA lid, initially exposing only a fraction of the intended core surface area to the dissolution medium, followed by complete lid dissolution. Slower initial drug release kinetics were expected in MV3 compared to MV1 due to the restricted access of dissolution media to the core through a 0.8 mm orifice, resulting in slower core hydration and subsequent drug release at early time points. Indeed, the drug release rate at 50% release (r50), measured as the gradient of a linear fit between 10% and 60% drug release [80], was 2.5 and 1.7 for MV1 and MV3, respectively, confirming the success of this design feature.

MV1 and MV3 exhibited faster release kinetics under USP 2 test conditions relative to their performance in preliminary drug release testing, which could be attributed to an increase in temperature from room temperature (preliminary tests) to 37 °C (USP 2) as well as a change in the agitation method from a magnetic stirrer to paddle apparatus. This temperature difference also contributed to a reduced solubility of dissolved gases and formation of air bubbles in the USP 2 dissolution media, which was not evident during preliminary tests. Air bubbles, originating from the medium, immobilized periodically on the surface of MV1 and MV2, could temporarily reduce surface area available for dissolution, contributing to the observed variability in individual release rates under USP 2 testing conditions. This argument is supported by the lack of variability in individual release rates from MV3 due to the air bubble removal protocol described in the methods section applied to all MV3-containing dissolution tests.

**Figure 8:**
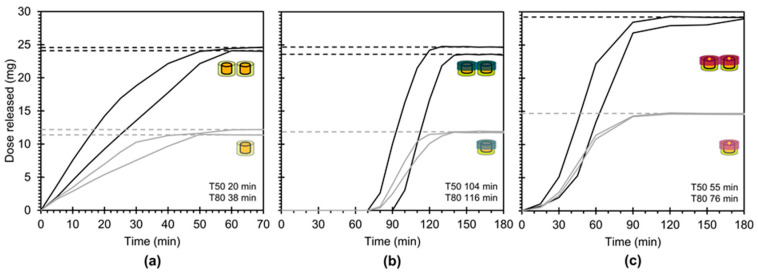
Dose released (mg) from dosage forms comprising two identical module variants: (**a**) MV1+MV1; (**b**) MV2+MV2; and (**c**) MV3+MV3. As a reference, solid grey lines indicate dose released from single module variants. Data is presented as the minimum and maximum of *n* = 2 samples. Mean T50 and T80 values for the dosage forms are displayed in the bottom right of each figure.

Figure 8 shows the dose released (mg) from the dosage form variants comprising two identical module variants namely MV1+MV1, MV2+MV2, and MV3+MV3, in Figure 8a–c, respectively.

The solid lines represent minimum and maximum drug release kinetics for the dosage form variants (black) and module variants (grey). Three unique release profiles are shown for the dosage form variants comprising two identical module variants, analogous to that of the single module variants. The dose released (mg) corresponds to the complete drug release (100%) in all cases. Furthermore, Figure 8 also confirms that, in the absence of interactions between individual modules, both dose and drug release kinetics from the dosage forms are a predictable net effect of the dose and drug release kinetics of their constituent modules in all cases.

Sources of variability for dosage forms constructed from MV1 and MV2 are as described for Figure 7. Additionally, lag time variability in MV2 can be related to sporadic print defects of the PVA lid as illustrated in Figure 5. The variability in dosage form variants constructed from MV3, which was not observed in the release from an individual MV3, could potentially have originated from adjacent placement of the module variants in the stationary basket, translating to differing hydrodynamics around each module variant. Due to the orifice of MV3 limiting the surface area available for hydration and matrix dissolution resulting in drug release, MV3 could exhibit a greater sensitivity to hydrodynamic fluctuations than MV1 or MV2.

**Figure 9:**
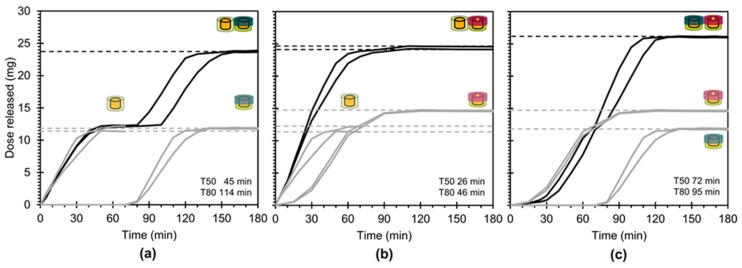
Dose released (mg) from dosage forms comprising two dissimilar module variants: (**a**) MV1+MV2; (**b**) MV1+MV3; and (**c**) MV2+MV3. As a reference, solid grey lines indicate dose released from single module variants. Data is presented as the minimum and maximum of *n* = 2 samples. Mean T50 and T80 values for the dosage forms are displayed in the bottom right of each figure.

Figure 9 shows the dose released (mg) from the dosage form variants comprising two different module variants namely MV1+MV2, MV1+MV3, and MV2+MV3, in Figure 9a–c, respectively.

The dose released (mg) corresponds to the complete drug release (100%) in all cases. As with the combination of identical module variants in Figure 8, Figure 9 confirms that a combination of dissimilar module variants to yield a dosage form also results in predictable, combined dose and drug release kinetics from the dosage form as a net effect of that of their constituent module variants. Since different module variants were combined, an additional three release profile variants were obtained on the dosage form level. T50 and T80 values in Figure 8; Figure 9 together demonstrate that a total of six release profile variants could be obtained from only three module variants, assuming the dosage form comprises two module variants. Similar drug release profiles at the dosage form level (e.g., Figure 8a compared to Figure 9b) suggest that overall variety in dosage form release profiles could benefit from a greater difference in individual module release profiles. Figure 9a demonstrates that drug release from each module occurs sequentially resulting in bimodal release kinetics compared to the remaining five dosage form variants where release from both modules commence simultaneously. In this study, sequential release is provided by the duration of the lag phase in one of the modules. However, sequential release can also be facilitated by alternative module designs and the order or pattern in which they are assembled in a final dosage form. This indicates that, based on the desired in vitro release profile on the dosage form level, for a particular drug product and/or therapeutic indication, the number of module variants assembled into a dosage form and their individual drug release kinetics can potentially be tailored to predictably yield multimodal release profiles, each with a controlled delivered dose, from the dosage form.

## 4. Discussion

### 4.1. Key Study Outcomes and Concept Potential

**Figure 10:**
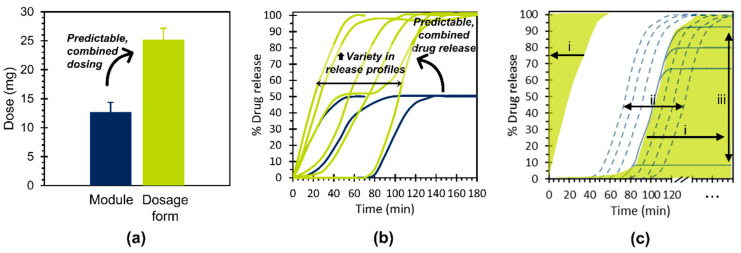
Summary of key demonstrated outcomes: (**a**,**b**); future potential of the concept to deliver increased product variety predictably using reconfigurable modularization: (**c**).

This study demonstrated how enhanced product variety for multifunctional individualization can be realized using reconfigurable modularization. Figure 10a,b depict key study outcomes. These include the use of reconfigurable modularization to:

- Independently achieve predictable, combined dosing and predictable, combined drug release kinetics for dosage forms based on their constituent modules and,
- Contribute to the enhanced product variety necessary for the provision of individualized therapy.

These study outcomes were underpinned by key differences between our proposed modular pharmaceutical product concept and conventional modular pharmaceutical product concepts, as summarized in Table 2.

In both the conventional and proposed modular product concepts, assembly may be physical assembly (e.g., gluing tablets, compression of bilayer tablets, and filling) [43,52,81], virtual assembly (e.g., through CAD models or additive deposition of modules to generate a product during additive manufacturing) [53,54,82,83], or hybrid assembly (e.g., compartmentalized devices, which can be filled with solids or liquids downstream) [44,84,85,86]. Importantly, prior to this demonstration of reconfigurable modularization, for conventional modular pharmaceutical product concepts, beyond customization, neither affordable customization nor the industrial adaption necessary for patient access to individualized therapies were sufficiently addressed. In fact, even compartmentalized designs with potential suitability for providing mutually independent dose, size, and release kinetics [44,53,54,61] are not yet adapted to a unified product–process–production context for addressing the provision of affordable variety and therefore patient access to individualized therapies. In response, in this study, product variety from a minimal number of modules is used as a simplified proxy for affordability. Whilst a more in-depth analysis of affordability in the realization of mass customization capability is highly desired, it is, albeit, beyond the scope of the present study. Accordingly, this study addressed a key challenge and eventual goal in promoting affordability during individualization, that is, to generate the maximum required number of finished product variants from a minimum number of module variants. For the purpose of demonstrating the proposed concept, although a low degree of product modularity was investigated (two modules in a dosage form), this principle was still evident through the use of reconfiguration to generate six unique dosage form release profile variants from only three module variants.

Beyond the demonstration of enhanced drug release flexibility independent of dose and the increase in total variety of release profiles, this concept provides additional opportunities for expanding flexibility while preserving affordability. Figure 10c illustrates that these include but are not limited to (i) increasing the total range of release profile variants, (ii) increasing the total number of release profile variants within a given range (fine-tuning a given release profile or obtaining new profiles), and (iii) enhancing dose flexibility independent of drug release.

(i) Increase the total range of dosage form drug release profiles: the release rate and mechanism is defined by the lid in our proposed product concept, either through the absence of the lid (MV1) or through the lid material and design (MV2 and MV3). Alternative lid materials or designs could therefore translate into alternative release kinetics or mechanisms for each module variant allowing either slower or faster kinetics than those demonstrated in this study. Modification of only the lid allows the balance between standardization and differentiation in the submodular components and the total number of module variants to remain fixed. This balance is one already established approach to simultaneously promote affordability (through standardization) and variety (through differentiation) during mass customization [31,36]. In addition, the degree of product modularity (number of modules assembled into a dosage form) demonstrated in this study was low, with a dosage form comprising only two out of three available module variant designs. Still, with the proposed product concept, reconfigurable assembly allowed an increased number of dosage form variants relative to module variants. This demonstration of the role of reconfiguration in promoting affordable variety through mass customization enables future exploration of the minimum number of product design modules and module variants that can enable a maximum number of dosage form/product variants. Increasing the number of module variants and/or the number of modules assembled into a dosage form (degree of product modularity) could also promote increased variety. However, the economic feasibility of an increased number of modules and module variants, beyond our demonstration, will depend in part on the volumes required of each relative to the overall gain in product variety.

(iia) Increase the total number of dosage form release profiles within a given range (through fine-tuning): Current academic research on individualized release predominantly utilizes a fine-tuning approach as a means to improve drug release flexibility, whereby modification of one release-determining structural feature allows tuning of drug release iteratively, on a continuous scale, and which confines variety in release profiles to variants of the same release profile shape or mechanism. Examples include modifying shell thickness in core–shell designs to prolong or shorten lag times [85,87], modifying porosity or the exposed surface area to obtain faster or slower kinetics with the same release profile shape [88,89,90], and so forth. This is also attainable with our product concept, for example, by modifying the exposed surface area of MV1 (as shown in Figure 6), modifying lid thickness of MV2 to achieve different lag times, or reducing orifice size of MV3 to achieve slower initial drug release kinetics. To support the latter, Table 1 indicates that 0.4 mm orifices can be printed with high precision. Subsequent translation to robust performance needs to be ensured. Beyond the smallest orifice size that can be printed with high precision, alternative approaches such as porous materials achieved though mixtures of water-insoluble polymers with water-soluble pore formers already exist [60,83,90,91,92]. This can not only provide fine-tuning as necessary for individualization but also expands the range of accessible release profiles as described above. One caveat, despite the technical potential for fine-tuning, is that the product variety–volume complexity challenge must be considered to assess economic feasibility while increasing the number of module variants.

(iib) Increase the total number of dosage form drug release profiles within a given range (through reconfiguration): Reconfiguration has been highlighted as a critical design feature of the proposed modular product concept to increase the number of dosage form variants without increasing the number of module variants that are designed and fabricated. Additionally, the dosage form was constrained in size to enhance patient acceptability. Our modular design was based upon a fixed target dosage form size of approximately 200 mm^3^ (corresponding to a standard 8 mm × 4 mm flat-faced cylindrical tablet) to remain within the currently recommended dimensions of marketed pharmaceutical tablets [93]. Since the modular product comprised only two modules, these were also designed to be below approximately 100 mm^3^ each. Multifunctionality requires that the tailoring of product features such as the dose and drug release be performed independently of each other and of dosage form size [3]. Furthermore, we have assumed that a dosage form comprises any two out of three available module variants. A dosage form consisting of a greater number of modules (whilst the total number of module variants remain the same) is one approach to obtain greater flexibility through reconfiguration. Since modular product concepts rely on the components of a dosage form being smaller than the dosage form itself due to assembly, the more modules make up a dosage form, the smaller the modules are required to be. Decreasing the size of modules could eventually be met with a trade-off in robustness since an increase in the specific surface area at a smaller size is often accompanied by an enhanced sensitivity to perturbations. A supporting example is the observed variability in individual release kinetics of MV1 and MV2 in this study, which was attributed to sensitivity to air bubbles in the test medium. Decreasing the module size is thus accompanied by strict requirements on precision fabrication and robust performance.

(i) and (ii) Modify both the range and number of dosage form release profiles through combined approaches: The total range of release kinetics selected in this study across the three module variants allowed demonstration of predictable, combined “monophasic” drug release and predictable, combined “biphasic” drug release. Since our results confirm that it is possible to construct dosage form release profiles predictably from their constituent module release profiles, tailoring the time gap between the release kinetics of one module variant and the next so that they are either more similar or less similar to each other can either achieve an overlap or reduce the overlap between the combined release profiles on a dosage form level. Tailoring the gap between module variant release kinetics together with modifying the number of modules that are reconfigured into a final dosage form can therefore result in multiphasic release profiles, if desired, for certain APIs and/or therapeutic indications.

(iii) Simultaneously tailor dose and drug release: Although this study demonstrated additive dosing from individual modules to final dosage forms, the delivered dose from the dosage form remained fixed (although not identical) between dosage form variants. Identical doses within individual module variants, if desired, could have been achieved through overfilling of MV1 and MV2 cores to match overfilling of MV3 cores. Alternatively, achieving scalable individualized dosing with modular dosage form design concepts at a fixed dosage form size was already addressed in our previous work [70]. As described above, maintaining acceptable dosage form sizes independent of the dose strength requires modules that are a fraction of the size of a conventional dosage form. However, it has been exemplified, through a modular product design concept for improving dose flexibility, that a large number of module variants are not necessary to yield high product variety, with only five module dose variants capable of providing 100 product dose variants [68]. Consequently, the proposed modular product concept can achieve simultaneous and independent tailoring of dose and drug release through spatial separation of the entire dose from the release-controlling functionalities allowing predictable modular dosing through modularization of the core [70] and predictable modular drug release.

### 4.2. Realization Challenges

Regarding individualized drug release, medical and biological knowledge gaps in defining target individual in vivo release profiles remain. Optimization of the product and its subsequent translation to in vivo performance was beyond the scope of this study. Nevertheless, our in vitro demonstration of an increased variety of predictable drug release kinetics provides a pre-emptive step towards realizing the overarching goals of individualized therapy, once these knowledge gaps are filled. Furthermore, although independent tailoring of dose and drug release through modularization was highlighted in this study, future explorations of modular product concepts for individualization will need to be extended to encompass additional product attributes such as the appearance, choice of API, composition, and sensory properties of a dosage form.

Robust functional realization and industrialization of our product concept and future product concepts with their individualized attributes will rely, in part, on processing technologies’ capabilities to fabricate parts reproducibly. Appropriate precision in FDM, HME, and melt molding was achieved in this study for adequate prototyping of the product concept. However, to expand potential utility of the concept and improve robustness of the module variants, alternative or modified formulations and processing strategies (e.g., injection molding) can be exploited. These can, for example, reduce core porosity and the contribution of air to orifice obstruction and variable release kinetics, achieve complete amorphization in the filament for drugs with poor aqueous solubility, or facilitate automation for increased throughput during scale-up and industrialization of the concept. The ability of the selected processes to achieve high precision in dose-controlling, release-controlling, and size-controlling structural features will remain critical.

Beyond product performance, unified integration with process and production will be required to promote access to affordable individualized therapy. Whilst initial explorations of product designs for individualization in a mass customization context have been performed [3,94], to this end, further aspects of the unified approach, beyond product modularization, remain to be explored (e.g., postponement and process flexibility) as enablers of a pharmaceutical mass customization paradigm. Even adaption of modular pharmaceutical product concepts into a pharmaceutical mass customization context still requires additional study areas, for example, establishment of appropriate affordability metrics, subsequent assessments of affordability, and the role of the position and type of module and product assembly steps in the pharmaceutical value chain, prior to their realization.

## 5. Conclusions

Through the provision of an increased variety of drug release profiles independent of the dose and size of the dosage form, a product concept for multifunctional individualization has been demonstrated. To the best of our knowledge this is the first demonstration that fulfills a crucial overarching requirement of pharmaceutical products for individualization, i.e., simultaneous and independent individualization of multiple product features in the context of a unified product–process–production approach for the provision of affordable variety.

The attainment of predictable, combined dosing and drug release with respect to the module variants from which a dosage form is constructed highlights the role of reconfigurable modularization in achieving predetermined dose and drug release accurately and predictably. In this regard, this study provides a first indication of the potential to reverse engineer drug release kinetics for a dosage form, from its composite module variants, based on a predetermined target release profile.

Furthermore, through reconfiguration during the final assembly stage (module variant to drug product variant) to manage product variety, this product concept is primed to translate already established key mass customization principles (process flexibility, modularization, and postponement) to the pharmaceutical value chain. Although the road to realization still requires optimization for robust performance, extended applicability to different APIs, and translation into clinical applicability, the dynamic product design presented here provides numerous additional opportunities for expanding flexibility, which remain to be harnessed prior to realization of affordable individualized therapy.

## Figures and Tables

**Table 1:** Mass and measured dimensions of submodular components governing dose and drug release.

**Submodular Component**	**Mean Mass (mg) ± SD**	**% RSD**	***n***	**Dose or Release Rate Determining**
PLA cups	29.72 ± 0.32	1.1	50	dose and release
Core (normal fill)	28.82 ± 1.04	3.6	20	dose
Core (overfill)	33.97 ± 1.42	4.2	10	dose
**Key Feature**	**Length (mm) ± SD**	**% RSD**	***n***	**Dose or Release Rate Determining**
Core diameter	3.81 ± 0.11	2.8	48	release
MV2 lid thickness	0.57 ± 0.04	6.4	16	release
MV3 orifice diameter (for 0.4 mm orifice)	0.39 ± 0.01	2.2	15	release

**Table 2:** Product modularization opportunities in the context of enhanced product variety during mass customization.

Key Descriptor	Conventional Modular Concepts	Proposed Modular Concept
**Purpose**	promotes process flexibility	promotes process flexibility and product variety
**Production context**	mass production—“economies of scale”	mass customization—“economies of scope”
**Product archetype(s)**	fixed assembly of components into products	reconfigurable assembly of components into products
**Product variant(s)**	typically achieved by iterative modifications of single initial product design	achieved by selection from multiple initial product designs
**Impact on product variety-volume complexity**	variety OR affordability achievable	variety AND affordability achievable
**Impact on product design**	no. of module variants > no. of product variants	no. of module variants < no. of product variants
